# Therapeutic potential of prolactin-releasing anti-dopaminergic agents in experimental diabetic retinopathy model

**DOI:** 10.1007/s00417-026-07173-9

**Published:** 2026-02-27

**Authors:** Kubra Peker, Ayse Ipek Akyuz Unsal, Ibrahim Meteoglu, Erol Erkan, Sinan Bekmez, Sayime Aydin Eroglu, Imran Kurt Omurlu, Turhan Dost, Buket Demirci

**Affiliations:** 1 Department of Ophthalmology, Faculty of Medicine, Aydin Adnan Menderes University, Aydin, Turkey; 2 Department of Pathology, Faculty of Medicine, Aydin Adnan Menderes University, Aydin, Turkey; 3 Department of Ophthalmology, Faculty of Medicine, University of Health Sciences, Dr. Behcet Uz Children Disease and Surgery Training and Research Hospital, Izmir, Turkey; 4 Department of Ophthalmology, Bakırçay University Çiğli Training and Research Hospital, Izmir, Turkey; 5 Department of Biostatistics, Faculty of Medicine, Aydin Adnan Menderes University, Aydin, Turkey; 6 Department of Medical Pharmacology, Faculty of Medicine, Aydin Adnan Menderes University, Aydin, Turkey

**Keywords:** Diabetic retinopathy, Gastrokinetics, Intraocular pressure, Prokinetics, Rational drug therapy, Schirmer test

## Abstract

*****What is known***:**

• Anti-dopaminergic agents (metoclopramide (MCP), trimethobenzamide (TMB), and domperidone (DOM)) are used for diabetic gastroparesis, but their effects on diabetic eye are not well studied.

*****What is new***:**

• These drugs do not affect intraocular pressure or tear production in diabetic rats.

• They increase retinal prolactin receptor expression and reduce VEGF and CD31 immunoreactivity, indicating modulation of angiogenesis-related markers in the diabetic retina.

• These findings may encourage future research to explore the potential role of prolactin-elevating antidopaminergic agents in diabetic retinopathy.

## Abstract (structured)

**Purpose:**

To investigate the ocular effects of prolactin elevation induced by anti-dopaminergic agents used for diabetic gastroparesis, including metoclopramide (MCP), trimethobenzamide (TMB), and domperidone (DOM), on intraocular pressure (IOP), tear production, and retinal angiogenesis-related markers in a streptozotocin-induced diabetic rat model.

**Methods:**

Fifty Wistar rats were randomly divided into five groups (*n* = 10 each): control, diabetes mellitus (DM), and DM treated with MCP, TMB, and DOM. Six weeks after diabetes induction, treatments were administered twice daily for two weeks. IOP and tear production (Schirmer test) were measured before enucleation. Retinal tissues were immunohistochemically analyzed for expression of the prolactin receptor, vascular endothelial growth factor (VEGF), and CD31. Staining intensity and the percentage of positive cells were semi-quantitatively scored, and mean values were compared among groups.

**Results:**

IOP and tear production showed no significant intergroup differences. Retinal staining was most prominent in the ganglion cell layer; therefore, this region was analyzed in detail. Prolactin receptor expression was significantly increased in all treatment groups compared with the diabetic and control groups (*p* < 0.05). VEGF and CD31 scores were elevated in the diabetic group but decreased with MCP, TMB, and DOM (*p* < 0.05).

**Conclusions:**

Prolactin-elevating antidopaminergic agents (MCP, TMB, and DOM) were associated with reduced immunoreactivity for VEGF and CD31 and increased expression of the prolactin receptor in the diabetic retina, without adverse effects on IOP or tear production. These findings warrant further investigation, including functional vascular assessments, to clarify their potential relevance in diabetic retinopathy.

## Introduction

Diabetes Mellitus (DM) is a growing global health concern, with associated complications such as nephropathy, gastropathy, neuropathy, and retinopathy [1]. Among these, diabetic retinopathy is a leading cause of vision impairment and blindness, affecting individuals in their productive years. It is a microvascular complication of DM and is characterized by damage to the retinal blood vessels. The pathogenesis of diabetic retinopathy is complex and involves upregulation of vascular endothelial growth factor (VEGF) and alterations in CD31, a marker of endothelial dysfunction. These changes, triggered by hyperglycemia, contribute to neovascularization and retinal damage. Anti-VEGF inhibitors are already in use for the treatment of diabetic retinopathy, and CD31 has also been suggested as a potential target for treating vascular permeability disorders [2]. Additionally, recent studies have explored the role of prolactin in the development and progression of diabetic retinopathy [3]. While traditionally associated with lactation, prolactin also influences glucose metabolism and angiogenesis. Prolactin overexpression leads to accumulation of vasoinhibin, a prolactin-derived end product, in the retina. This accumulation inhibits pro-angiogenic molecules such as VEGF, inactivates nitric oxide synthase (NOS) enzymes, and ultimately hinders retinal vasopermeability [4]. Thus, prolactin and its product, vasoinhibin, emerge as promising molecules for the prevention and treatment of diabetic retinopathy and diabetic macular edema by modulating angiogenesis and vasopermeability through various pathways.

Pharmacological interventions in diabetic patients may inadvertently affect prolactin levels. For example, gastroparesis, a common gastrointestinal complication in this population, is frequently treated with antidopaminergic agents such as metoclopramide (MCP), trimethobenzamide (TMB), and domperidone (DOM) [5]. These antidopaminergic medications work by blocking dopaminergic D2 receptors and are commonly used to relieve symptoms like nausea, indigestion, and bloating associated with gastroparesis [6, 7]. However, this blockade prevents dopamine’s inhibitory effect on prolactin release, resulting in elevated prolactin levels. While these drugs effectively manage gastrointestinal symptoms, their effects on prolactin levels raise concerns about their potential impact on retinal health, particularly in diabetic patients.

Beyond their impact on retinal vasculature, diabetes and medications used to manage its complications can also affect other ocular structures and functions. Diabetic patients are at increased risk of developing dry eye syndrome and experiencing changes in intraocular pressure (IOP), both of which are vision-threatening complications [8, 9]. Given the potential impact of antidopaminergic drugs on prolactin levels and the established links between diabetes and changes in the ocular surface and IOP, it is crucial to investigate the broader ocular effects of these medications in diabetic patients. Therefore, this study aims to investigate the ocular effects of antidopaminergic drugs (MCP, TMB, and DOM), specifically focusing on their impact on retinal vasculature, dry eye parameters, and IOP, in a streptozotocin (STZ)-induced diabetic eye model.

## Materials and methods

This study was approved by the Aydin Adnan Menderes University Animal Experiments Local Ethics Committee (HADYEK 64583101/2024/04). Fifty adult male Wistar Albino rats were housed in transparent polycarbonate cages under controlled environmental conditions, including room temperature of 24–27 °C, relative humidity of 40–70%, adequate ventilation, and a 12 h light/12 h dark cycle. Standard commercial rat laboratory diet and tap water were provided ad libitum throughout the study. All animals were monitored daily by a veterinarian and the research team.

An experimental STZ-induced diabetes model, widely accepted for diabetic retinopathy studies, was used [10]. Rats aged 3–4 months were randomly divided into five groups of 10 each, and baseline body weights were recorded. Four groups received a single intraperitoneal injection of STZ (Streptozotocin, Sigma, Interlab, Istanbul, Turkey) at 60 mg/kg, dissolved in physiological saline. Seventy-two hours after injection, blood glucose levels were measured from tail vein samples using a glucometer (Clever check, Istanbul, Turkey). Rats with blood glucose levels > 250 mg/dL were considered diabetic. Body weights and blood glucose levels were reevaluated at the end of the sixth week. Drug treatments were then administered for two weeks.

### Experimental groups

- Group 1 (Control; *n* = 10): No medications or treatments are given.
- Group 2 (DM; *n* = 10): STZ-induced diabetes, no further treatment for 8 weeks.
- Group 3 (DM + MCP; *n* = 10): STZ-induced diabetes followed by intramuscular metoclopramide (MCP) 2 mg/kg twice daily for two weeks (Metpamid^®^, Sifar Pharmaceuticals Trade and Industry Inc., Istanbul, Turkey).
- Group 4 (DM + TMB; *n* = 10): STZ-induced diabetes followed by intramuscular trimethobenzamide (TMB), 4 mg/kg twice daily for two weeks (Emedur^®^, Opella Healthcare Consumer Health Inc., Istanbul, Turkey).
- Group 5 (DM + DOM; *n* = 10): STZ-induced diabetes followed by oral domperidone (DOM) 10 mg/kg twice daily for two weeks (Motilium^®^, Sanofi Pharmaceuticals Industry and Trade Inc., Istanbul, Turkey).

### Intraocular pressure and tear pruduction assessment

**Fig. 1:**
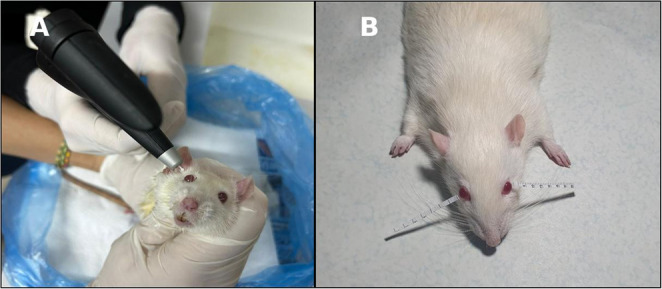
(**A**)Measurement of intraocular pressure using a rebound tonometer in a rat under general anesthesia. (**B**) Representative photograph of the Schirmer test procedure in a rat eye performed during the present study. Standardized Schirmer strips are placed in the lateral one-third of the lower eyelid margin to assess tear production. After five minutes, the strips are removed, and the length of the wetted area (in mm) is measured

At the end of the eighth week, intraocular pressure was measured in all rats under ketamine (50 mg/kg) and xylazine (5 mg/kg) anesthesia using a noninvasive rebound tonometer (iCare^®^ Tonovet tonometer, Finland) (Fig. 1A).

Schirmer’s test was subsequently performed to evaluate tear production (Fig. 1B). Standardized Schirmer’s strips were gently placed in the lateral one-third of the lower eyelid margin, as described in previous rodent dry eye studies [11]. After five minutes, the strips were removed, and the length of the wetted area was measured in millimeters.

### Tissue collection and histopathological examination

Following the ocular assessments, rats were sacrificed under deep anesthesia. Both eyes were enucleated and fixed in 10% neutral buffered formalin. After routine tissue processing, tissues were embedded in paraffin, and 4-µm-thick sections were obtained.

Sections were stained with hematoxylin and eosin (H&E) and examined under a light microscope (BX51, Olympus, Tokyo, Japan) at magnifications of x10, x20, and x40. Light microscopic changes were evaluated, particularly in the cornea, retina, choroid, and sclera.

### Immunohistochemical staining

Immunohistochemical staining was performed using a DAKO Autostainer Universal Staining System (Autostainer Link 48, DAKO, Glostrup, Denmark). Four-micrometer-thick sections were mounted on positively charged slides, deparaffinized in xylene, and dehydrated through a graded ethanol series. Antigen retrieval was performed at 96 °C for 40 min in citrate buffer (10 mM/L, pH 6.9) using a PT Link system.

Sections were incubated for 60 min with the primary antibodies:

- anti-CD31 (Clone JC70A, Mouse anti-human, RTU, IR 610, DAKO).
- anti-VEGFA (MAb JH1121, Invitrogen, Waltham, MA, USA, 1:200 dilution).
- anti-Prolactin Receptor (Ab-1, Clone B6.2; Thermo Fisher Scientific, Fremont, CA, USA, 1:400 dilution).

An automated streptavidin-biotin immunoperoxidase technique (EnVision Flex, K8000; DAKO) was used. Diaminobenzidine (DAB) served as the chromogen to visualize antibody binding, and sections were counterstained with hematoxylin. After dehydration through an ascending alcohol series and clearing in xylene, slides were mounted with coverslips using balsam. All immunohistochemical stainings were performed simultaneously for all experimental groups, using the same antibody batches and identical incubation times and conditions. Tissues known to exhibit positive staining served as positive controls, while negative controls were obtained by replacing the primary antibody with normal rabbit IgG.

### Semi-quantitative evaluation

All sections were evaluated by light microscopy by a single experienced pathologist (I.M.) who was blinded to the experimental groups. Immunostaining intensity was scored from 0 (no staining) to 3 (strong staining), and the percentage of positively stained cells was recorded. A semi-quantitative score was calculated for each section by combining staining intensity and distribution. Immunostaining was assessed primarily in the retinal ganglion cell layer, where staining was most prominent; staining observed in other retinal layers was considered non-specific and excluded from analysis. For each group, the total number of positively stained areas across all examined microscopic fields was divided by the number of rats in the group. This calculation provided a staining score for statistical comparison between groups.

### Statistical analysis

The Kolmogorov-Smirnov test was used to assess the normality of the data distribution. Non-normally distributed data were analyzed using the Mann-Whitney U test or the Kruskal-Wallis test. Categorical data were compared using chi-square tests. Results were reported as mean ± standard deviation, median (25-75th percentile), or frequency (%). Statistical significance was set at *p* < 0.05.

## Results

The mean Schirmer test results and intraocular pressure values for the groups are shown in Table 1. There was no statistically significant effect of anti-dopaminergic drugs on lacrimation and intraocular pressure (*p* = 0.10 and *p* = 0.131, respectively).

**Table 1:** Mean Schirmer test and intraocular pressure (IOP) values of the groups

Schirmer test (mm)	Control (*n* = 16)	DM (*n* = 14)	DM + MCP (*n* = 16)	DM + TMB (*n* = 18)	DM + DOM (*n* = 18)	*p*
	6.50 ± 2.0	5.81 ± 1.44	6.17 ± 1.83	5.41 ± 1.04	6.73 ± 1.59	0.10
IOP (mm Hg)	5.56 ± 1.31	4.35 ± 1.33	4.93 ± 1.48	5.38 ± 1.85	5.83 ± 2.03	0.131

*n* Number of the rats. *DM* Diabetes mellitus, *DM + MCP* Diabetes mellitus treated with Metoklopramid, *DM + TMB* Diabetes mellitus treated with Trimetobenzamid, *DM + DOM* Diabetes mellitus treated with Domperidon

**Fig. 2:**
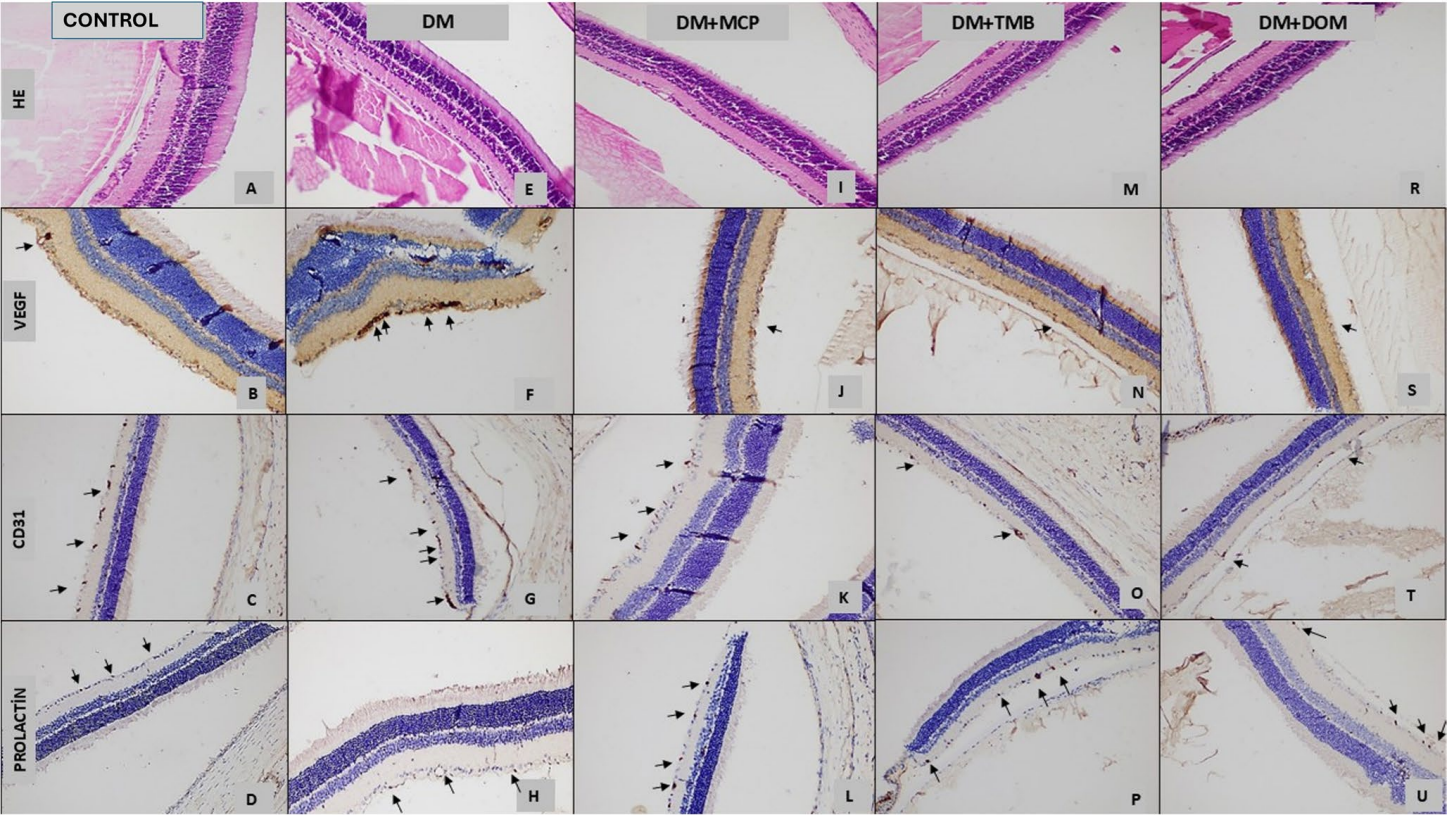
Immunohistochemical staining of retinal sections in all groups. (**A**-**D**) Control group. H&E staining (**A**), VEGF (**B**), CD31 (**C**), and prolactin receptor (**D**) immunostaining (x200). (**E**-**H**) DM group. H&E staining (**E**), VEGF (**F**), CD31 (**G**), and prolactin receptor (**H**) immunostaining (x200). (**I**-**L**) DM + MCP group. H&E staining (**I**), VEGF (**J**), CD31 (**K**), and prolactin receptor (**L**) immunostaining (x200). (**M**-**P**) DM + TMB group. H&E staining (**M**), VEGF (**N**), CD31 (**O**), and prolactin receptor (**P**) immunostaining (x200). (**R**-**U**) DM + DOM group. H&E staining (**R**), VEGF (**S**), CD31 (**T**), and prolactin receptor (**U**) immunostaining (x200). Arrows indicate positively stained areas within the ganglion cell layer

Because significant staining was primarily observed in the retinal ganglion cell layer, only this area was analyzed in detail (Fig. 2). Staining in other parts of the eye was considered non-specific. As described in the immunohistochemical evaluation section of the method, a score was calculated for each group by dividing the total staining score by the number of rats in the group, based on staining intensity for Prolactin, VEGF, and CD31.

**Fig. 3:**
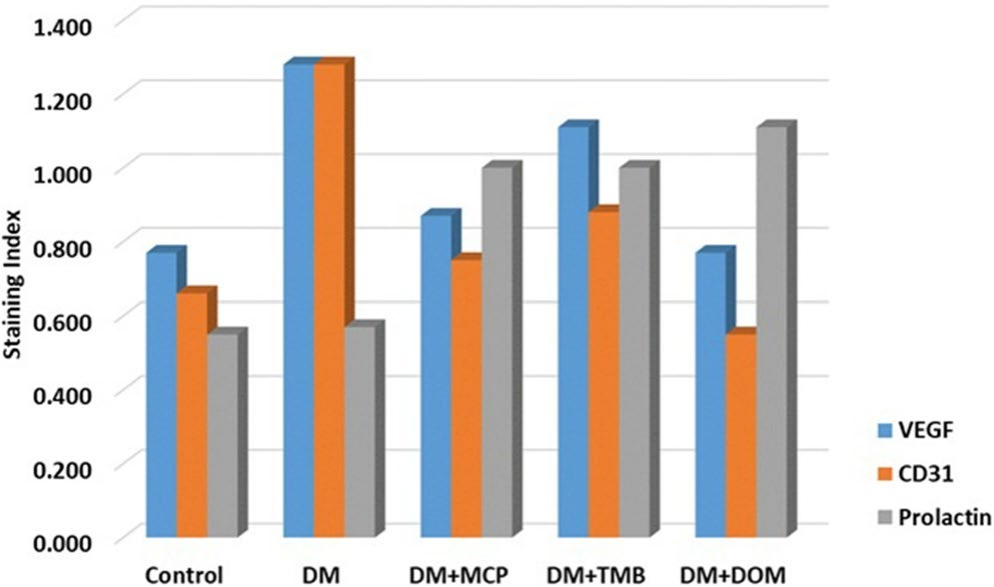
Bar graph showing immunohistochemical staining scores for VEGF, CD31, and the prolactin receptor in retinal ganglion cell layers across all experimental groups

Immunohistochemical staining scores for the prolactin receptor, VEGF, and CD31 in all experimental groups are shown in Table 2. As shown in Fig. 3, the bar graph illustrates the distribution of these scores in the retinal ganglion cell layer across the experimental groups.

**Table 2:** Immunohistochemical staining scores of prolactin receptor, vascular endothelial growth factor (VEGF), and CD31 in all experimental groups

Prolactin	Control (*n* = 16)	DM (*n* = 14)	DM + MCP (*n* = 16)	DM + TMB (*n* = 18)	DM + DOM (*n* = 18)	*p*
	0.56	0.57	1.00	1.00	1.11	0.004
VEGF	0.78	1.28	0.88	1.11	0.78	0.006
CD31	0.67	1.29	0.75	0.89	0.56	0.012

*n* Number of the rats. *DM* Diabetes mellitus, *DM + MCP* Diabetes mellitus treated with Metoklopramid, *DM + TMB* Diabetes mellitus treated with Trimetobenzamid, *DM + DOM* Diabetes mellitus treated with Domperidon. Note: Staining was evaluated in the retinal ganglion cell layer, where significant immunoreactivity was observed. Other retinal regions were considered non-specific

The prolactin receptor scores were as follows: in the control group, 0.56; in DM, 0.57; and in the treatment groups with MCP, TMB, and DOM, the scores increased to 1.0, 1.0, and 1.11, respectively (*p* = 0.004). The increase in prolactin receptor expression was significant in all rats in the treatment groups (MCP + TMB+DOM, *p* = 0.001) (Fig. 3).

While VEGF staining in the control group scored 0.78, the DM group’s score increased to 1.28. In the treatment groups, the scores decreased to 0.88 for MCP, 1.11 for TMB, and 0.78 for DOM (*p* = 0.006) (Fig. 3).

The CD31 scores followed a similar pattern to VEGF. Induction of diabetes nearly doubled the CD31 score to 1.29. Treatment with MCP, TMB, and DOM reduced the scores to 0.75, 0.89, and 0.56, respectively (*p* = 0.012) (Fig. 3).

## Discussion

Diabetic retinopathy and diabetic macular edema are among the leading causes of vision loss in adults worldwide [12]. The growing number of diabetic patients significantly increases the incidence of these eye diseases, and estimates suggest that the prevalence of diabetic retinopathy and diabetic macular edema is expected to rise by 55% by 2030 as the number of individuals with diabetes increases globally [13].

Failure to properly control ocular angiogenesis is the cause of diabetic retinopathy and other vasoproliferative retinopathies [14]. Chronic hyperglycemia leads to the loss of pericytes and endothelial cells, increasing retinal vasopermeability and the accumulation of extracellular fluid and hard exudates, thereby impairing vision when the macula is affected.

In the present study, we observed a statistically significant decrease in VEGF scores in the treatment groups compared with the DM group. CD31 scores showed a similar trend. Diabetes induction nearly doubled the CD31 score, while we found a significant reduction in CD31 scores with MCP, TMB, and DOM treatments. Additionally, we found that prolactin receptor scores were higher in the treatment groups.

Giving these drugs for two weeks was only sufficient to increase prolactin receptors on retinal ganglion cells. Meanwhile, VEGF and CD31 expression were limited, and even these mediators tended to decrease. VEGF increase, as an underlying pathology of diabetic retinopathy, promoted new capillary formation in the endothelium, which could be demonstrated by CD31 staining.

These findings align with an earlier study on the benefits of prolactin and its effects on the retina [15]. This study used sulpiride, another dopamine antagonist, and osmotic minipumps to deliver prolactin, aiming to increase both prolactin and its fragment vasoinhibin in the diabetic retina [15]. The researchers showed that when prolactin levels were elevated through sulpiride administration or exogenous prolactin, retinal vasoinhibin levels increased, along with a reduction in retinal hyperpermeability. This study was conducted after 4 weeks of STZ induction [15]. In our study, rats were observed for 6 weeks to develop the complication, to better mimic real-life conditions. Additionally, sulpiride treatment was found to reduce VEGF-induced retinal hemorrhages [15].

Vascular endothelial growth factor is a key driver of vasopermeability in diabetic macular edema and diabetic retinopathy [15]. A clinical trial investigated oral levosulpiride for 8 weeks and reported that patients with diabetic macular edema and diabetic retinopathy had improved visual and structural outcomes, mediated by intraocular upregulation of vasoinhibin and downregulation of VEGF [16, 17].

Levosulpiride is considered a prokinetic agent that causes hyperprolactinemia as a side effect [18]. Studies have reported that levosulpiride increases levels of vasoinhibin, a prolactin fragment, in the vitreous, and vasoinhibin is an effective inhibitor of retinal hyperpermeability in rodent models [4, 19]. Similarly, in our study, we found a significant increase in prolactin receptor expression in retinal tissue, which may reflect activation of prolactin-related signaling pathways, potentially including vasoinhibin-mediated mechanisms, that contribute to suppression of VEGF and CD31.

Consistent with these observations, experimental studies in rodent models have shown that hyperprolactinemia leads to vasoinhibin accumulation in the retina and reduces both VEGF-induced and diabetes-induced retinal vascular permeability [3]. Moreover, vasoinhibin gene transfer has been shown not only to prevent but also to reverse excessive retinal vasopermeability and oxygen-induced retinal angiogenesis, providing strong biological support for the role of prolactin-related pathways in regulating retinal vascular homeostasis [20].

Each protein domain in CD31 plays distinct roles in cells during the progression of inflammatory diseases [21]. Decreased levels of VEGF and CD31 have been shown to reduce angiogenesis and improve various diseases associated with diabetic retinopathy [22]. CD31, a member of the immunoglobulin superfamily, is expressed at high levels at the boundaries between platelets, monocytes, neutrophils, and T cell subsets. Homophilic CD31 interactions are crucial for maintaining an intact endothelium and also play a key role in the transendothelial migration of CD31-positive leukocytes [23]. CD31 and VEGF, essential mediators of angiogenesis, contribute to the progression of diabetic retinopathy. In our study, we demonstrated that increased systemic prolactin also leads to a rise in the number of prolactin receptors in the retina and suppresses mediators such as VEGF and CD31, which are involved in the pathogenesis of diabetic retinopathy.

However, the present study provides preliminary mechanistic evidence that prolactin elevation modulates angiogenic markers in the diabetic retina. In clinical practice, anti-VEGF agents are widely used to treat diabetic macular edema and neovascular age-related macular degeneration, where angiographic imaging clearly demonstrates a reduction in retinal vascular leakage following VEGF inhibition. In this context, the observed downregulation of VEGF and CD31, in parallel with increased prolactin receptor expression, suggests that prolactin elevation may indirectly contribute to reduced retinal vascular permeability. Nevertheless, this study was not designed to directly assess functional vascular endpoints such as retinal vascular permeability, blood-retinal barrier integrity, pericyte loss, leukocyte recruitment, or perfusion abnormalities. We did not perform pericyte-specific staining, leukostasis assays, or quantitative vascular permeability measurements (e.g., Evans Blue or FITC-dextran). Therefore, while the observed molecular and immunohistochemical changes support a potential modulatory role of prolactin elevation on angiogenic pathways involved in diabetic retinopathy, these findings should be interpreted as preliminary rather than definitive evidence of vascular protection. Further studies incorporating functional vascular permeability assays, pericyte markers, and leukocyte-endothelial interaction analyses are required to confirm these findings and better define the therapeutic potential of prolactin-elevating agents in diabetic retinopathy.

One of the strengths of this study is the comparison of three prolactin-releasing medications within the same experimental setup. Additionally, we showed that prolactin receptors increase in retinal tissue, not just in the serum, contrary to previous research that confirmed a sulpiride-induced rise in serum prolactin [15]. However, it is important to further assess the long-term safety and therapeutic potential of these drugs.

The results also provide important insights into the ocular safety profile and potential clinical applications of the agents under investigation. None of the antidopaminergic drugs significantly affected tear production or intraocular pressure. These medications are commonly used as antiemetics in the perioperative period, including after ocular surgeries performed under general anesthesia, such as glaucoma surgery. Preserving tear function and maintaining stable intraocular pressure is critical in patients with pre-existing ocular surface disease or glaucoma. Therefore, the absence of adverse effects on lacrimation and intraocular pressure observed in this study may represent an additional clinical benefit when these agents are used as antiemetics, compared with other antiemetic drugs with known anticholinergic properties, such as scopolamine or promethazine [24].

In conclusion, MCP, TMB, and DOM demonstrated a favorable ocular safety profile in this experimental diabetic retinopathy model, with no significant effects on intraocular pressure or tear production. These findings provide preliminary mechanistic evidence that prolactin-elevating antidopaminergic agents may modulate angiogenesis-related pathways in the diabetic retina, as reflected by decreased VEGF and CD31 expression and increased prolactin receptor expression in retinal ganglion cells. However, these results should be interpreted in light of the study’s methodological scope, which did not include functional vascular assessments. Further studies evaluating retinal vascular permeability, blood-retinal barrier integrity, and long-term structural and functional retinal vascular outcomes are required before these agents can be considered therapeutic options for diabetic retinopathy.

## Data Availability

Data available on request from the authors. The data supporting the findings of this study are available from the corresponding author, AIAU, upon reasonable request.
